# Salicylates

**Published:** 1890-06-14

**Authors:** 


### THERAPEUTICS.

### SALICYLATES.

Some physicians, notably Dr. Charteris, are of opinion that the artificial salicylic acid obtained by Kolbe's process, *i.e.*, by the passage of carbonic acid through phenol (the so-called carbolic acid), is necessarily inferior to that obtained from the gaultheria, or the natural acid, but there is no doubt that the former is apt to contain impurities from which the latter is free; and that if this could be avoided the artificial and less costly would, as in the case of cocaine, alizarine, vanillin and other synthetic products, be in every respect equal to, because identical with the corresponding natural forms. Dr. B. Fischer, in the Pharmac.-Zeitung discusses the question exhaustively, and shows that the defects and perhaps the dangers attaching to artificial salicylates are mainly owing to the use of impure carbolic acid for their manufacture. Commercial phenol always contains an intermixture of cresol, to which is due its well-known tendency to assume a red colour when kept in solution and exposed to the air and light, When cresol is present cresotinic acid is formed. An excess of the caustic soda used for the separation of the salicylic acid in the form of its soda salt, and also too low a temperature during the process favour the formation of parabenzoic acid, while if on the other hand the temperature be too high, the carbonic acid reacts on the salicylic already formed, producing oxisophthalic acid, and, lastly, the oxidation of an alkaline solution of salicylic acid developes a brown colouring matter of undetermined composition. Paroxybenzoic acid, however, may be easily separated, in virtue of its far readier solubility in water, and Demme has shown that of the three forms of cresotinic acid, the orthocresotinic alone can be looked on as dangerous. It betrays its presence by the imperfectly crystalline form and faint carbolic odour of the sample. Solutions of sodic salicylates later inevitably develope a brown colour, but when freshly prepared should be perfectly clear.

